# Beyond DNA damage: 3D tumor models and the integrin mechanobiology of radioresistance

**DOI:** 10.1186/s13046-026-03803-6

**Published:** 2026-08-05

**Authors:** Nils Cordes

**Affiliations:** 1 OncoRay-National Center for Radiation Research in Oncology, Faculty of Medicine Carl Gustav Carus, Technische Universität Dresden, PF 41, Fetscherstrasse 74 , 01307 Dresden, Germany; 2 Institute of Radiooncology - OncoRay, Helmholtz-Zentrum Dresden-Rossendorf (HZDR), Dresden, 01328 Germany; 3 German Cancer Research Center, German Cancer Consortium, Partner Site Dresden, Heidelberg, 69120 Germany; 4 National Center for Tumor Diseases (NCT), Partner Site Dresden, German Cancer Research Center (DKFZ), Heidelberg, 69192 Germany

**Keywords:** 3D tumor models, Radioresistance, Tumor microenvironment, Extracellular matrix, Integrins, Precision oncology, Patient-Derived Organoids

## Abstract

Despite major advances in radiation delivery, clinical outcomes remain constrained by tumor biology rather than technology. Conventional radiobiology has relied on reductionist two-dimensional (2D) systems that fail to capture the spatial, mechanical, and multicellular organization of tumors. Three-dimensional (3D) tumor models now resolve radiation response as a mechanobiological process coordinated across the extracellular matrix (ECM), adhesion signaling, cytoskeleton, and nucleus. This review focuses on a specific and we argue, underappreciated intersection: how 3D models expose integrin-mediated mechanotransduction as a determinant of the DNA damage response (DDR) and therapy resistance epitomized by cell adhesion-mediated radioresistance (CAM-RR) - an organizing principle we frame as the ECM-integrin-nucleus axis. We first delineate which model classes resolve which layer of this biology, distinguishing effects of three-dimensional organization from those of defined ECM-integrin signaling, and we treat the underlying mechanobiology quantitatively rather than descriptively. We then develop a mechanistic framework linking ECM architecture and stiffness to integrin-RTK crosstalk, cytoskeletal tension, LINC-mediated force transfer, and chromatin-dependent DNA repair, in which radiosensitivity emerges as a property of tissue context. From a translational standpoint, 3D models enable functional, radiation-specific assessment of context-dependent radiosensitivity and of mechanically targeted radiosensitization, while their integration with quantitative imaging and computational approaches further supports biomarker-guided and adaptive treatment strategies. We close with testable predictions that this framework generates, intended to guide the next phase of biology-driven radiation oncology.

## Background

Radiotherapy has achieved extraordinary technological precision [1–8], yet treatment outcomes remain fundamentally constrained by tumor biology. Even tumors with similar histology and genomic alterations frequently exhibit markedly divergent responses to identical radiation regimens, highlighting the profound influence of tumor ecosystem heterogeneity [9–12]. It reflects complex and dynamic biological processes that are not fully captured by current diagnostic approaches, thereby limiting the ability to predict treatment response and optimize patient-specific therapy.

Traditionally, radiobiology has focused on DNA double-strand breaks (DSBs) as the principal cytotoxic lesions induced by ionizing radiation [13–16]. While this paradigm has been instrumental in elucidating DNA damage response (DDR) pathways, it does not sufficiently explain the heterogeneity of clinical outcomes. Tumors are not collections of isolated malignant cells but spatially organized multicellular ecosystems in which biochemical signaling, tissue mechanics, and extracellular architecture collectively determine stress adaptation and therapy response [17]. Cellular responses to radiation are therefore controlled by a combination of biochemical factors, including oxygen and nutrient availability, cellular composition such as immune and stromal infiltration, and biomechanical cues such as extracellular matrix (ECM) architecture and tissue stiffness [18–24]. Together, these parameters regulate survival, proliferation, metabolism, and stress signaling, thereby influencing radiosensitivity in a context-dependent manner.

While two-dimensional (2D) cell culture models have provided important mechanistic insights, they inadequately reproduce force transmission, ECM-dependent signaling organization, and spatially coordinated adaptive responses observed in tumors in vivo. These key aspects of tumor biology such as spatial signaling gradients, chromatin organization, and multicellular coordination are insufficiently represented in conventional 2D systems but are more faithfully recapitulated in three dimensionally (3D) tumor models. There is a large variety of 3D tumor models such as multicellular spheroids, ECM-based cultures, patient-derived organoids, assembloids, and engineered platforms.

The biological basis for these differences lies in the dimensionality of cell-matrix engagement itself. In 2D, cells adhere through a single ventral plane and adopt a flattened, non-physiological morphology with artificially polarized integrin engagement, whereas in 3D they experience multidirectional ECM contacts, restored apical-basal polarity, three-dimensional cell-cell junctions, and spatial confinement. These features reorganize focal adhesion architecture, cytoskeletal tension, and nuclear shape, and they generate the diffusion-limited gradients of oxygen, nutrients, and pH that are absent on rigid plastic [25–32]. It is this combination of altered adhesion topology and emergent gradients, rather than three-dimensionality per se, that drives the distinct transcriptional, epigenetic, and DNA repair phenotypes seen in 3D [33, 34].

Previous reviews have largely surveyed organoid technologies or the cellular composition of the tumor microenvironment. Cancer mechanobiology is itself a mature field; the contribution we aim to make is therefore narrower and, we believe, sharper, namely to connect three converging strands that are usually treated separately: the use of 3D models as controlled experimental systems, integrin-mediated mechanotransduction, and radiation-related DDR. Viewed together, biomechanical organization emerges not as one more microenvironmental variable but as an upstream regulator that biases how radiation damage is sensed, processed, and repaired.

Insights from 3D systems have shifted the conception of radiation response from a cell-intrinsic consequence of DNA damage toward an emergent property of the tumor ecosystem [35–37]. Within this view, ECM composition, integrin-mediated adhesion, mechanotransduction, and chromatin organization interact across spatial and temporal scales to regulate survival and DNA repair, with CAM-RR as the paradigmatic example of how tumor cell-ECM interactions sustain survival under genotoxic stress [38]. Because the network is robust and redundant, disrupting a single node, such as one integrin, is often insufficient, and the relative weight of each mechanism varies across tumor entities, regimens, and spatial contexts.

To orient readers less familiar with mechanobiology, we state briefly why integrins, rather than any single microenvironmental factor, occupy the center of the framework developed here, before their signaling is dissected in detail below. Integrins are heterodimeric (αβ) transmembrane receptors that form the principal molecular interface between the ECM and the cell interior [38–40]. Three properties make them the organizing node of the ECM-integrin-nucleus axis. First, they attach to defined ECM ligands, such as collagens, fibronectin, and laminins, with subunit-specific selectivity, so that matrix composition is perceived as distinct adhesion signals. Second, they signal bidirectionally, converting intracellular cues into altered ligand binding (‘inside-out’) and, conversely, transducing extracellular mechanical and biochemical cues into intracellular biochemical and cytoskeletal responses (‘outside-in’). Third, through focal adhesions and the physically continuous actin-LINC machinery, they transmit force from the ECM to the nuclear envelope, where it is converted into chromatin states that co-determine DNA repair capacity and pathway choice [41–44]. Integrins are therefore located upstream of, and are mechanically coupled to, the cytoskeletal, nuclear, and DNA repair events that shape radiation response, and it is integrin-dependent adhesion signaling that operationally defines CAM-RR [33, 34]. They are not the only mechanotransducers, and YAP/TAZ, Piezo channels, and caveolar dynamics contribute in context-dependent ways [45–47]. However, integrins represent the ligand-specific, adhesion-determining, and pharmacologically targetable hub where these signals converge; they therefore serve as the anchor point for the subsequent mechanistic and translational sections.

We synthesize current evidence into a unifying framework in which radiation response is coordinated through an integrated ECM-integrin-nucleus axis. Within this axis, ECM architecture is translated via integrin-mediated adhesion into signaling that engages RTKs, cytoskeletal dynamics, and chromatin-dependent DNA repair. Although we survey the full spectrum of 3D models, our objective is not to catalogue platforms but to use them as instruments for dissecting this axis; each model class is therefore judged by the specific layer it can resolve. From a translational perspective, the framework provides a basis for functional precision radiotherapy through improved patient stratification, biomarker development, and rational, mechanically informed combination strategies. 

### 3D radiobiology as a paradigm shift

**Fig. 1:**
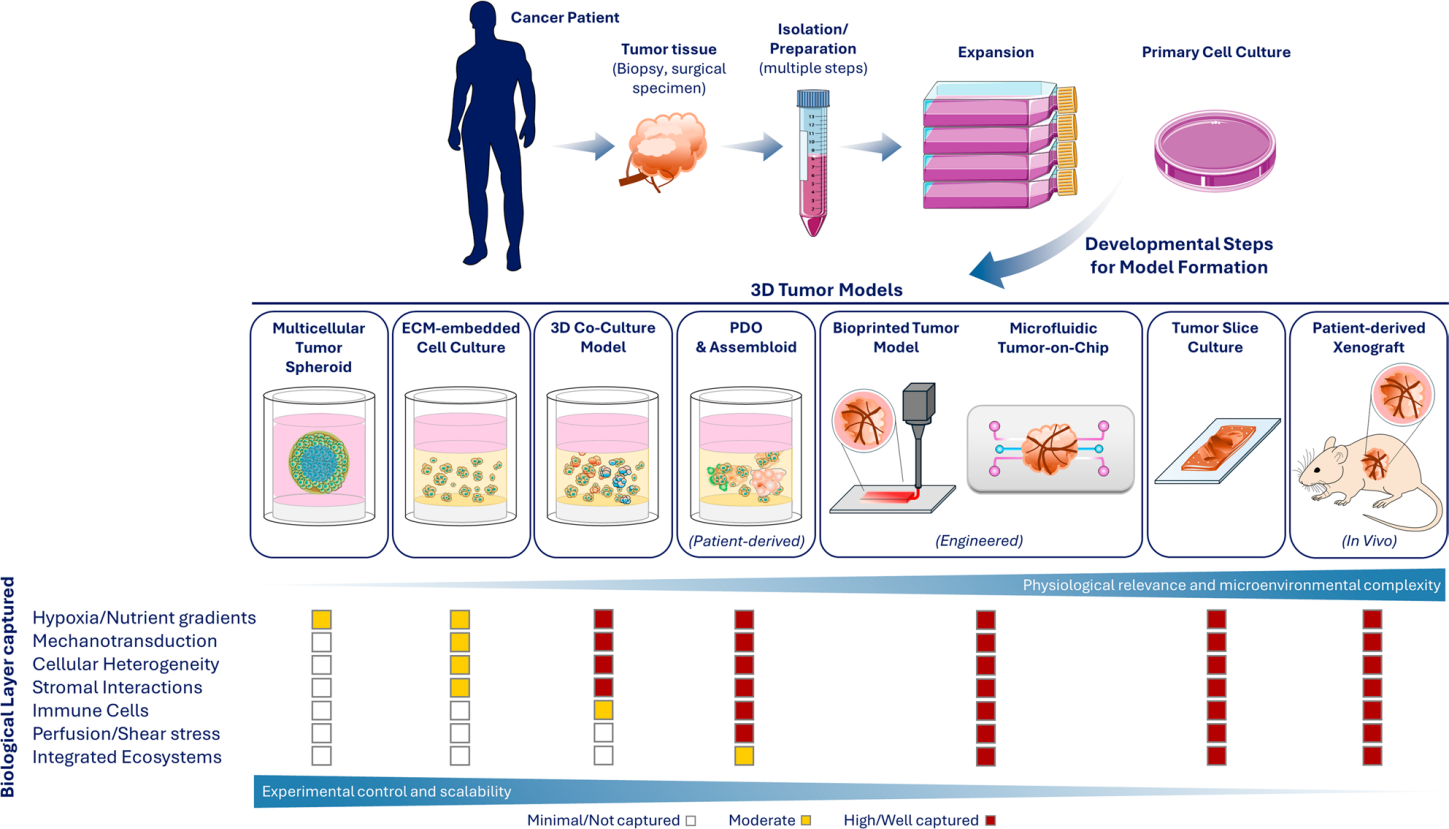
Spectrum and functional hierarchy of 3D tumor models in radiation oncology. Schematic overview of commonly used 3D tumor model systems arranged according to increasing biological complexity and physiological relevance. Multicellular spheroids recapitulate gradients of oxygen, nutrients, and proliferation, enabling investigation of hypoxia-associated radioresistance and radiation penetration. ECM-based 3D cultures restore integrin-mediated adhesion signaling and mechanotransduction, providing platforms to study cell adhesion-mediated radioresistance (CAM-RR) and stiffness-dependent effects. Co-culture systems incorporate stromal and immune components to model microenvironment-driven resistance mechanisms. Patient-derived organoids preserve tumor heterogeneity and genetic architecture, enabling functional assessment of patient-specific radiosensitivity. More advanced systems, including organoid-based assembloids and PDO-immune co-cultures, integrate fibroblasts, immune cells, and endothelial cells to reconstruct multicellular tumor ecosystems and dynamic tumor–stroma–immune interactions. Additional platforms, such as bioprinted tissues and microfluidic tumor-on-chip systems, allow precise control of spatial organization, ECM composition, perfusion, and treatment response. Tumor slice cultures and patient-derived xenografts (PDX) provide complementary ex vivo and in vivo validation systems with high physiological relevance. Together, these models form an integrated toolkit for dissecting context-dependent radiation responses across multiple levels of tumor complexity

3D tumor systems enable cell growth within physiologically and pathologically relevant ECM environments characterized by defined viscoelasticity, spatial organization, biomechanical signaling, and microenvironmental gradients that fundamentally co-regulate chromatin organization [28, 30, 33, 48–61]. As summarized in Fig. 1; Tables 1 and 2, these systems reproduce key aspects of tumor organization [62–64]. Consistent with this, cells cultured in 3D exhibit molecular and phenotypic characteristics that more closely resemble in vivo tumors, including transcriptional, epigenetic, and (phospho)proteomic profiles [56, 65–68].

**Table 1:** Three-dimensional tumor modeling approaches used in radiobiology and radiation oncology

3D approach	Materials / technical components	Pros for radiobiology research	Limitations	References
Multicellular tumor spheroids	Low-adhesion plates, hanging drop systems, ultra-low attachment plates; tumor cell lines or primary cells	Simple, reproducible, scalable; generates oxygen, nutrient, and proliferation gradients; suitable for hypoxia-driven radioresistance and radiation penetration studies	Limited ECM representation; lacks stromal and immune components; reduced architectural complexity	[69, 70]
ECM-embedded 3D cultures (matrix-based models)	Collagen I, laminin-rich matrices (e.g., Matrigel), fibronectin; tumor cells embedded in hydrogels	Recapitulates ECM adhesion, integrin signaling, and mechanotransduction; ideal for studying CAM-RR and stiffness-dependent effects	Batch variability; limited control of matrix composition; often lacks multicellular complexity	[57, 71, 72]
3D co-culture systems	Tumor cells + fibroblasts, endothelial cells, immune cells; ECM scaffolds or hydrogels	Models for tumor-stroma-immune interactions; enables study of stromal signaling, immune modulation, and microenvironment-driven radioresistance	Increased complexity; reduced reproducibility; difficult standardization and interpretation	[73, 74]
Patient-derived Organoids (PDO)	Basement membrane extracts (e.g., Matrigel); patient tumor cells; specialized growth factor media	Preserves tumor heterogeneity, architecture, and genetics; enables functional precision radiotherapy and patient-specific radiosensitivity testing	High cost and technical demands; limited stromal, immune, and vascular components	[75–77]
Patient-derived organoid (PDO) / Immune co-cultures	Patient tumor organoids, autologous TILs or PBMCs, ECM hydrogels (e.g., Matrigel)	Recapitulates patient-specific immune-tumor crosstalk; models ‘immune exclusion’ and exhaustion; ideal for testing radio-immunotherapy synergy.	High technical complexity; requires fresh patient tissue; difficult to standardize for high-throughput.	[78–80]
Organoid-based assembloids	Combination of different patient-derived organoids or with stromal (e.g., fibroblasts), and/or endothelial cells; ECM hydrogels (e.g., Matrigel, collagen); co-culture or sequential assembly strategies	Reconstructs multicellular tumor ecosystems; enables study of tumor-stroma-immune interactions; captures dynamic cell-cell and cell-matrix signaling; highly relevant for investigating microenvironment-driven radioresistance	High technical complexity; limited standardization; variability in cellular composition; challenges in scalability and reproducibility	[81–83]
Bioprinted tumor models	Bioinks (collagen, gelatin, alginate, synthetic hydrogels); 3D bioprinter; tumor + stromal cells	Precise spatial control; tunable ECM composition and stiffness; enables vascular-like structures and architecture-specific radiation studies	Technically demanding; high cost; limited maturation and standardization	[84–86]
Microfluidic tumor-on-chip systems	Microfluidic devices, ECM hydrogels, perfusion systems; tumor and stromal cells	Dynamic control of perfusion, oxygen, and shear stress; models vascular responses and treatment-induced microenvironment changes	High technical complexity; low throughput; limited standardization	[87, 88]
Tumor slice cultures (ex vivo tissue slices)	Fresh patient tumor tissue slices; precision-cut tissue sections; short-term culture systems	Preserves native tissue architecture, ECM, vasculature, and immune context; highly relevant for ex vivo radiation response validation	Limited lifespan; variability between samples; low scalability	[89, 90]
Patient-derived xenografts (PDX, in vivo)	Implantation of patient tumor tissue into immunocompromised mice	Maintains tumor heterogeneity and architecture; enables in vivo validation of radiation response and combination therapies	Time-consuming; expensive; species differences; limited immune system representation	[91]

**Table 2:** Conceptual hierarchy of tumor models - complexity, relevance and applications

Model	Biological complexity	Physiological/ Clinical relevance	Best suited to study		Additional key radiation research applications	Typical readouts (radiation-specific)
			CAM-RR	RTK crosstalk		
Multicellular tumor spheroids	Low-medium	Moderate	❌	⚠ฏ	Drug penetration, basic mechanistic radiobiology	Spheroid volume/growth kinetics; Dissociated clonogenic assay (loses spatial context); Hypoxia staining (Pimonidazole/HIF-1alpha); Proliferation (Ki67); DNA repair kinetics (γH2AX/53BP1 foci)
ECM-embedded 3D cultures (matrix-based models)	Medium	Moderate	✅	✅	Integrin signaling, ECM effects	CAM-RR index (clonogenic survival in matrix); DNA repair kinetics (γH2AX/53BP1 foci); Integrin signaling (p-FAK, p-Src); Nuclear shape/Chromatin density
3D co-culture systems	Medium-high	High	✅	✅	Tumor-stroma-immune interactions; microenvironment-driven resistance	Spatial immune exclusion (e.g. T-cell infiltration distance); CAF activation (α-SMA); Paracrine signaling (Cytokine/Chemokine arrays); Juxtacrine survival effects; DNA repair kinetics (γH2AX/53BP1 foci)
Patient-derived Organoids (PDO)	High	High	✅	✅	Personalized radiotherapy; patient-specific radiosensitivity testing	Organoid formation efficiency (OFE); 3D cell viability (CellTiter-Glo 3D); Dose-response curves (GR50); Histopathological fidelity to original tumor; DNA repair kinetics (γH2AX/53BP1 foci)
Patient-derived organoid (PDO) / Immune co-cultures	Very high	High (emerging)	✅	✅	Personalized radiotherapy; patient-specific radiosensitivity testing and tumor-immune cell interactions	Organoid formation efficiency (OFE); 3D cell viability (CellTiter-Glo 3D); Dose-response curves (GR50); Histopathological fidelity to original tumor; DNA repair kinetics (γH2AX/53BP1 foci) Spatial immune exclusion (e.g. T-cell infiltration distance)
Organoid-based assembloids	Very high	High (emerging)	✅	✅	Personalized radiotherapy; patient-specific radiosensitivity testing and tumor-stroma-immune interactions; microenvironment-driven resistance	Organoid formation efficiency (OFE); 3D cell viability (CellTiter-Glo 3D); Dose-response curves (GR50); Histopathological fidelity to original tumor; DNA repair kinetics (γH2AX/53BP1 foci)
Bioprinted tumor models	Very high	High (emerging)	✅	⚠ฏ	Spatial architecture, ECM mechanics, customizable radiation studies	Spatial heterogeneity mapping; ECM deposition/remodeling; Long-term tissue architecture stability; Regional radiation sensitivity; DNA repair kinetics (γH2AX/53BP1 foci)
Microfluidic tumor-on-chip systems	Very high	High (emerging)	⚠ฏ	✅	Dynamic radiation response; perfusion and vascular effects	Shear stress response; Real-time migration/invasion tracking; Perfusion/Oxygenation levels; Microvascular integrity and radiation-induced leakiness; DNA repair kinetics (γH2AX/53BP1 foci)
Tumor slice cultures (ex vivo tissue slices)	Very high	Very high	✅	✅	Ex vivo validation; tissue-level radiation response	Ex vivo TCD50 (tissue control dose); Spatial DDR in native niche; Multiplexed IHC of intact tissue architecture; Metabolomic profiling; DNA repair kinetics (γH2AX/53BP1 foci)
Patient-derived xenografts (PDX, in vivo)	Highest	Highest	✅	✅	Preclinical validation; therapy testing in vivo	Tumor growth delay (TGD); TCD50; In vivo imaging (PET/CT/Bioluminescence); Metastatic frequency after local irradiation; DNA repair kinetics (γH2AX/53BP1 foci)

✅ highly suitable, ⚠ฏ limited suitable, ❌ not suitable

**Fig. 2:**
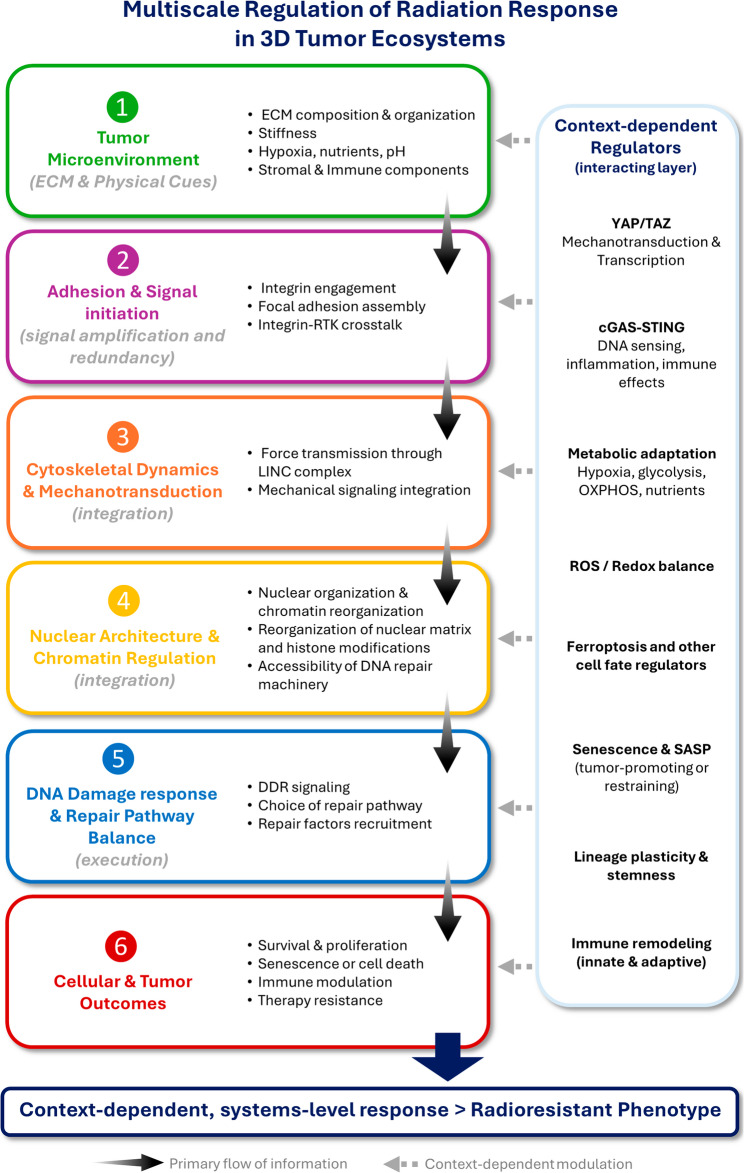
Schematic representation of radiation response as an integrated mechanobiological process emerging from reciprocal interactions between extracellular architecture, adhesion signaling, cytoskeletal dynamics, and nuclear organization

A major contribution of 3D models is the ability to resolve how microenvironmental context determines the radiation response and, thus, presents as an integration of multiple signaling layers (Fig. 2) [34, 71, 92–98]. These insights have led to the recognition of microenvironment-driven resistance mechanisms [81, 99, 100], such as CAM-RR, which are not adequately captured in conventional systems. In addition, evidence also implicates metabolic adaptation, immune-mediated signaling, redox regulation, and lineage plasticity as important contributors to context-dependent radioresistance.

Different classes of 3D tumor models address distinct aspects of tumor biology. Multicellular tumor spheroids represent a robust and scalable platform to study hypoxia-associated radioresistance by reproducing gradients of oxygen and proliferation [82, 92]. ECM-based models, including collagen- and laminin-rich matrices, enable mechanistic investigation of cell-matrix interactions and mechanotransduction [33, 83, 101]. Patient-derived organoids preserve tumor heterogeneity and genetic alterations, providing clinically relevant systems for functional assessment of therapy response [50, 102, 103]. More complex platforms, such as co-culture systems, assembloids, bioprinted tissues, and microfluidic tumor-on-chip technologies, incorporate stromal and immune components and allow dynamic modeling of tumor-microenvironment interactions [104–112]. Together, these models form a continuum of increasing biological complexity, enabling selection of appropriate systems depending on the specific experimental or translational question (Fig. 1; Tables 1 and 2).

These classes differ fundamentally in whether the effects they capture arise from three-dimensional organization itself or from defined ECM-integrin signaling. Scaffold-free spheroids reproduce gradients, cell-cell contacts, and tissue-scale architecture but lack a defined exogenous ECM, whereas matrix-embedded cultures, organoids, and engineered tissues additionally supply the integrin ligands, stiffness, and mechanical cues required to study mechanotransduction. This distinction has direct consequences: commonly used basement-membrane extracts (Matrigel) are very soft (on the order of tens to a few hundred pascals) and lie more than an order of magnitude below the stiffness of most solid tumors, so the absence of mechanical tunability is itself a source of artefact. Engineered 2D substrates of defined, tunable stiffness therefore remain valuable and, for strictly mechanical questions, can be more faithful than a soft 3D gel. These systems are best regarded not as discrete categories but as an inter-compatible continuum that can be combined to add complexity; microfluidic formats in particular add control and scale-down rather than constituting a distinct three-dimensional class [53, 113, 114].

The value of 3D models extends beyond improved fidelity. By integrating biochemical and mechanical cues across spatial scales, they allow radiosensitivity to be interpreted as a property of interacting networks. Greater complexity improves the capacity to capture such behavior but also raises challenges of standardization, reproducibility, and scalability. A parallel repertoire of 3D normal tissue models addresses therapy-limiting normal tissue toxicity summarized and reviewed elsewhere [115–117].

From a translational perspective, 3D tumor models provide a platform for functional precision radiation oncology. By enabling direct assessment of context-dependent radiosensitivity, these systems facilitate the identification of microenvironmental vulnerabilities and support the development of biomarker-guided and combination treatment strategies. While no single model fully recapitulates tumor complexity, integrative use of complementary 3D systems is increasingly recognized as essential for bridging mechanistic insights and clinical application.

In the following sections, we discuss how specific components of the tumor microenvironment (TME) including tissue architecture, multicellular organization, ECM composition, and nuclear regulation interact to determine radiation response within the ECM-integrin-nucleus axis.

### Multicellular organization, tissue architecture, and ECM as determinants of radiation response

Tumors are mechanically and spatially heterogeneous tissues composed of genetically and phenotypically distinct cellular populations. In contrast to the structured architecture of normal tissues, tumor organization is typically disordered, characterized by loss of polarity, irregular vascularization, and inefficient perfusion [118–120]. These architectural abnormalities generate pronounced gradients of oxygen, nutrients, and metabolites, leading to hypoxic and metabolically stressed niches that critically influence radiosensitivity [121–125]. Consequently, radiation response is not solely determined by intrinsic cellular properties but emerges from the spatial organization of cells within the tumor and their local microenvironment.

A central component shaping this organization is the ECM, which, beyond serving as a structural scaffold, functions as a dynamic network of structural proteins (collagens, elastin), glycoproteins (fibronectin, laminins, others such as nidogen, tenascin, vitronectin), and proteoglycans (such as aggrecan, decorin, perlecan), and glycosaminoglycans (hyaluronic acid, chondroitin sulfate, dermatan sulfate, heparan sulfate, keratan sulfate) that provide mechanical support and biochemical cues [37, 126, 127]. Tumors often exhibit highly heterogeneous and altered ECM composition and stiffness due to increased collagen deposition, crosslinking, and remodeling by tumor cells themselves as well as cancer associated fibroblasts (CAFs) [128–130]. ECM properties influence the effects of radiation through various mechanisms and vice versa. Hypoxic and poorly perfused areas reduce the effectiveness of radiation [131], while the ECM composition can affect drug delivery and the infiltration of immune cells [132–135]. Furthermore, ECM remodeling dynamically alters the mechanical and biochemical environment during therapy. Radiation itself can induce ECM remodeling in a variety of tumor types, observed in fibrosis or enhanced desmoplasia, i.e. excessive deposition of ECM protein in the extracellular space [136–139].

Contrary to common assumptions, tumor stiffness is not uniform and not always elevated [140]. While increased stiffness is frequently associated with fibrosis and tumor progression in primary breast cancer, pancreatic cancer, glioblastoma, and other cancer types, decreased stiffness has also been observed in the same and other malignant tumors as well as at different sites of metastasis [124, 141–147]. Reduced stiffness suggests tissue destruction, necrosis, or altered stromal composition [148]. The simultaneous presence of increased and decreased stiffness within the same tumor, i.e., ECM heterogeneity, is thus a direct reflection of the general tumor heterogeneity.

Beyond the direction of these changes, their magnitude and molecular nature matter, and 3D models increasingly capture them quantitatively. Normal soft tissues are compliant (typically below ~ 1–2 kPa), whereas desmoplastic breast and pancreatic carcinomas can stiffen to ~ 5–20 kPa or more, predominantly through collagen deposition, lysyl-oxidase-mediated crosslinking, and fiber alignment rather than a uniform elevation in matrix density [42, 149]. Critically, ECM composition is not a reliable surrogate for stiffness, and the two need not act in the same direction: increasing stiffness and crosslinking generally promote integrin clustering, focal adhesion maturation, FAK/PI3K-AKT signaling, and we propose a relative shift toward rapid non-homologous endjoining (NHEJ), whereas the high matrix density accompanying fibrosis simultaneously impairs diffusion of oxygen, drugs, and antibody-based integrin inhibitors, a dual effect that may help explain why integrin-targeting agents have underperformed in stiff, dense tumors. The mechanical environment is also dynamic: ionizing radiation can acutely soften the matrix by cleaving collagen, reducing stiffness and adhesion [150], while the subsequent fibrotic response progressively stiffens the tissue through myofibroblast activation and excess ECM deposition [151, 152]. Radioresistance mechanobiology is thus best understood as a biphasic, time-dependent process, which further motivates non-invasive stiffness read-outs detected by approaches such as magnetic resonance elastography (MRE) to identify novel candidate biomarkers [153, 154]. These techniques offer the possibility of observing these biomechanical changes in vivo and could be combined with other radiological methods like radiomics to gain even more comprehensive insights [155]. Overall, stiffness should be considered a context-dependent parameter that integrates various aspects of tumor biology. The ECM represents a key regulator of tumor behavior and a crucial factor in treatment response. Taken together, the ECM is not merely a structural component but a dynamic regulator of radiosensitivity that integrates mechanical, biochemical, and spatial cues.

Beyond cell-ECM interactions, integrin-dependent signaling also influences cell-cell adhesion and tissue cohesion through crosstalk with cadherins and other adhesion systems [145–147]. These mechanisms regulate collective cell behavior, including migration, survival signaling, and coordinated responses to stress. Following irradiation, such multicellular interactions can enhance tumor resilience, as damaged cells receive survival signals and structural support from neighboring cells. In parallel, paracrine and autocrine signaling networks, cancer stem cell niches, metabolic reprogramming, and interactions with stromal and immune components further determine the tumor microenvironment and contribute to therapy resistance [37, 148].

Collectively, these observations support the concept that the ECM functions not merely as a passive adhesive structure but as a biophysical signaling hub. These aspects are of paramount importance because especially clinical studies generally do not allow for close and comprehensive monitoring of tumor behavior during and after therapy.

### Nuclear architecture and chromatin organization

The nucleus is mechanically coupled to the cytoskeleton through the ECM-integrin-actin-nuclear envelope axis and associated linker complexes [29, 34, 156–158]. These structures transmit mechanical cues from the extracellular environment to the nucleus and chromatin. This is particularly relevant because tumor cell behavior is generally co-regulated by cell-ECM and cell-cell interactions. Likewise, these structures serve as critical determinants of cellular radiosensitivity. Proteins involved are cytoskeletal connectors such as actin and plectin, core LINC complex components like nesprin1-4 (SYNE1-4), and Sun proteins (SUN1/2) [157, 159–165]. The cytoskeleton is anchored via these structures to the nuclear lamina formed by lamin A/C and B-type lamins, which further connect to chromatin through LEM-domain proteins (emerin, LAP2) and chromatin-tethering factors such as BANF1 and HP1 [166–168]. This structural coupling regulates nuclear shape, stiffness, and positioning, influencing higher-order chromatin organization and accessibility of DNA to damage and repair.

Experimental evidence supports the existence of direct mechanical coupling between the ECM and chromatin. Mechanical forces applied to integrins rapidly propagate through cytoskeletal filaments to the nucleus, inducing nuclear deformation, nucleolar repositioning, and reorganization of chromatin-associated structures [43, 169]. This phenomenon is consistent with the concept of a structural continuum, in which extracellular signals are integrated through both biochemical pathways and mechanotransduction to modulate nuclear function [170, 171]. At the epigenetic level, ECM-driven changes in cell morphology and cytoskeletal tension control chromatin organization governed by histone modifications and chromatin remodeling complexes. In contrast to 2D, cells embedded in 3D matrices typically exhibit a different nuclear shape, more compact chromatin and altered epigenetic landscapes resembling those in tissues [34, 172–174]. The chromatin status influences both the induction and repair of DNA damage. Dense heterochromatin can reduce DNA accessibility to radiation-induced free radicals and repair enzymes, whereas open euchromatin is more vulnerable but may allow rapid repair [175, 176]. Therefore, nuclear organization likely appears as an important determinant of radiosensitivity [177–179]. Experimental comparisons between 2D and 3D growth conditions reveal differences in the number, distribution, and resolution kinetics of radiation-induced DNA damage foci with foci in 3D cultures being fewer and more equally distributed to eu- and heterochromatic areas [34, 180]. Cell-ECM interactions play a great part in the regulation of these processes through numerous downstream effectors, which modulate nuclear processes by regulating cell cycle checkpoints, transcriptional programs, and DNA repair pathway choice [181, 182]. Disruption of adhesion signaling or cytoskeletal tension leads to nuclear softening, chromatin decondensation, and increased radiation-induced cell death like apoptosis, underscoring the functional importance of cell-matrix interactions in maintaining radioresistant phenotypes [44, 183]. Altogether, available evidence supports the concept that (i) chromatin functions as a mechanosensitive platform that integrates extracellular forces into stable epigenetic states, (ii) the LINC complex appears to function as a critical conduit linking ECM-derived mechanical signals to chromatin organization and transcriptional regulation, (iii) remodeling of the nuclear envelope in cancer cells modifies both mechanical properties and genome organization, facilitating therapy resistance but also local infiltration and distant metastasis. Importantly, chromatin and its posttranslational modifications provide mechanical memory, a fundamental mechanism eliciting persistent tumor phenotypes independent of genetic mutations [184–187].

A further consequence of mechanical coupling is that nuclear deformation can directly generate DNA damage, linking architecture to genome integrity independently of radiation. In confining 3D environments, the nucleus undergoes large deformations that transiently rupture the nuclear envelope, causing mislocalization of repair factors, replication stress, and DNA breaks; the frequency of rupture rises as confinement increases and as lamin levels fall [188–190]. This mechanically induced damage, together with stiffness-dependent control of chromatin compaction, means that the same ECM cues that bias repair pathway choice also modulate the substrate on which radiation-induced breaks must be repaired, a dependency that only matrix-embedded or confining 3D systems can reveal.

In the TME, where ECM composition and stiffness vary spatially, such mechanoregulation contributes to heterogeneous radiosensitivity within the same lesion. These observations challenge the classical view that radiation response is governed predominantly by DNA damage induction and repair and instead support a model in which nuclear organization, dynamically regulated by cell adhesion and mechanical cues, determines how damage is sensed, processed, and repaired. Within the ECM-integrin-nucleus axis, nuclear organization seems to form the final integrative layer at which mechanical and biochemical signals converge to regulate DNA repair. Targeting components of this axis therefore provides a mechanistically grounded strategy for radiosensitization.

To place these adhesion-dependent mechanisms in context, it is worth recalling the classical, cell-intrinsic determinants of radiosensitivity. Ionizing radiation kills cells predominantly through DSBs, whose repair is governed by the DDR: ATM, ATR, and DNA-PKcs signaling, checkpoint activation, and the choice between NHEJ and homologous recombination (HR), modulated by intrinsic radiosensitivity, oxygenation, and proliferative state [13–16]. CAM-RR and the integrin-RTK network do not replace this cell-intrinsic view but constitute a microenvironmental layer superimposed upon it, biasing checkpoint control and repair pathway choice through adhesion-dependent signaling. Dissecting this layer requires systems that reconstitute cell-ECM engagement; consequently, matrix-embedded 3D cultures, organoids, and engineered tissues, but not scaffold-free spheroids or 2D monolayers, are the platforms in which CAM-RR can be faithfully recapitulated.

### Integrin signaling and crosstalk between integrins and growth factor receptors

**Fig. 3:**
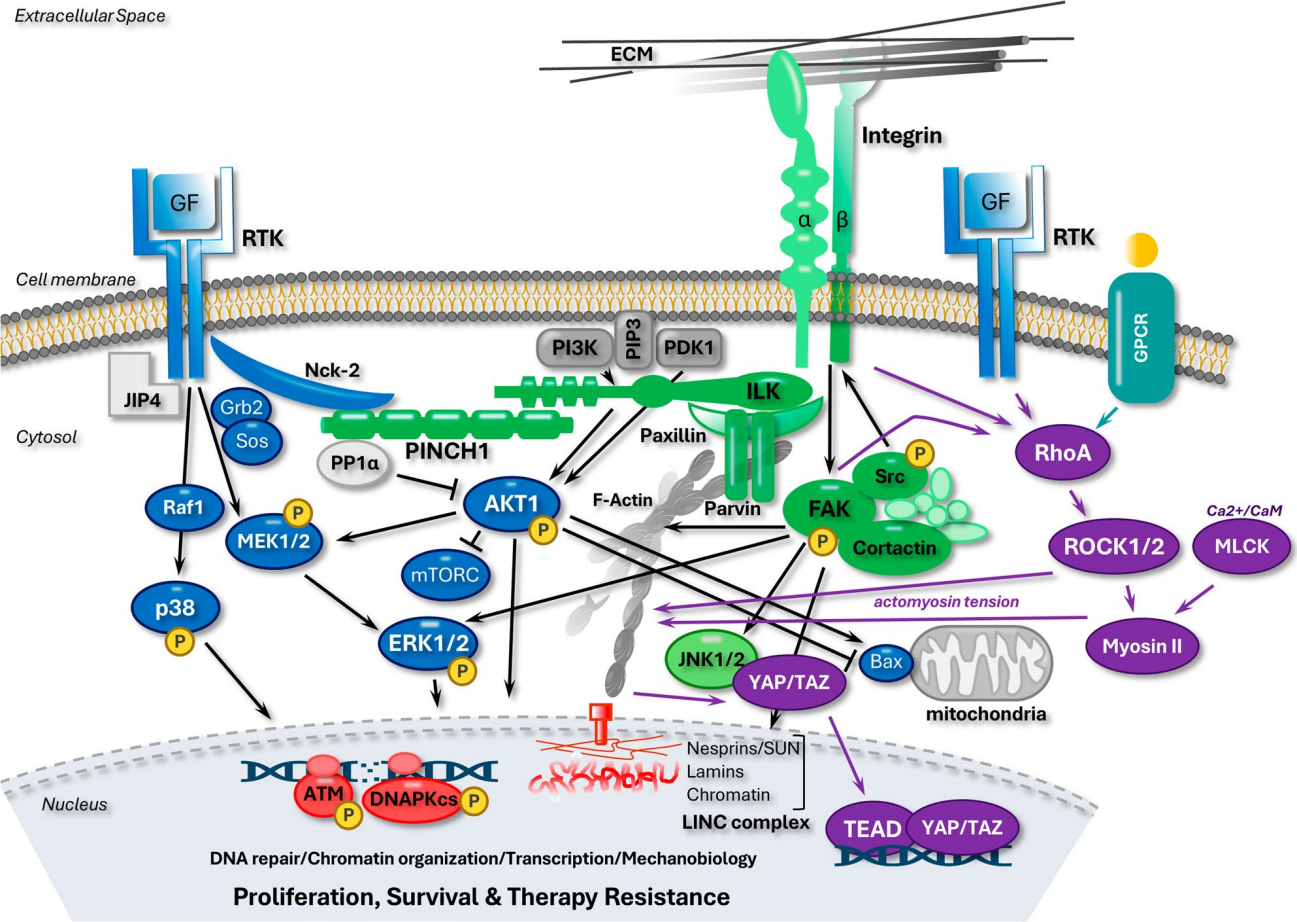
The ECM-integrin-nucleus axis as a mechanobiological framework driving radioresistance. Integrin-associated signaling networks integrate extracellular matrix (ECM) architecture, growth factor receptor signaling, actomyosin contractility, and chromatin regulation to coordinate the radiation response. Engagement of α/β integrins by ECM ligands such as fibronectin, collagen, and laminin nucleates focal adhesion assembly. Subsequently, adhesome and signaling proteins, including FAK, Src, cortactin, paxillin, and parvin, are recruited. The integrin-linked ILK-PINCH1-parvin (IPP) complex, together with the adaptor Nck-2 and the phosphatase PP1α, connects adhesion receptors to the actin cytoskeleton and to receptor tyrosine kinases (RTKs), promoting ligand-independent RTK activation and downstream PI3K-PIP3-PDK1-AKT1/mTORC and Grb2-Sos-Raf1-MEK1/2-ERK1/2 signaling, alongside the stress-activated p38 and JNK1/2 (JIP4) modules. These pathways support proliferation and survival, suppress mitochondrial (Bax-dependent) apoptosis, and, together with ATM- and DNA-PKcs-dependent signaling, enhance repair of radiation-induced DNA double-strand breaks. Components highlighted in violet denote the mechanobiology layer, comprising actomyosin contractility and YAP/TAZ-mediated mechanotransduction. Mechanical and chemical cues acting through integrin/FAK adhesions, RTKs, and G-protein-coupled receptors (GPCRs) activate the small GTPase RhoA and its effector kinase ROCK1/2, while Ca^2+^/calmodulin (Ca^2+^/CaM)-activated MLCK provides a parallel, RhoA-independent input. ROCK1/2 and MLCK converge on myosin II to generate actomyosin tension along F-actin stress fibers. This cytoskeletal tension is transmitted to the nucleus through the LINC complex (Nesprins/SUN/lamins), shaping chromatin organization and DNA repair pathway utilization, and concomitantly drives nuclear translocation of the transcriptional co-activators YAP/TAZ and their engagement of TEAD transcription factors. Collectively, these convergent biochemical and mechanical inputs regulate DNA repair, chromatin organization, transcription, and mechanobiological control, establishing spatially organized signaling hubs that underlie cell adhesion-mediated radioresistance (CAM-RR) and adaptive therapy resistance

We propose that integrin-mediated adhesion signaling represents an important organizing axis of adhesion-dependent stress response (Figs. 2 and 3). In this model, here referred to as the ECM-integrin-nucleus axis, ECM architecture is translated via integrins and associated adhesome complexes into coordinated signaling outputs that involve reciprocal crosstalk with RTK activation, cytoskeletal tension, and nuclear organization (Figs. 2 and 3) [39, 191–193]. These processes converge on chromatin accessibility, chromatin state, and DNA repair efficiency, thereby contributing to radiosensitivity as an emergent property of tissue context rather than a purely cell-intrinsic trait.

**Table 3:** Integrin-associated signaling networks and context-dependent mechanisms of cell adhesion-mediated radioresistance

Signaling module	Representative components	Role in CAM-RR / radiation response	Key downstream effects	Representative tumor contexts	Therapeutic implications	References
ECM-integrin adhesion	β1, αvβ3, αvβ5, α6β4 integrins	Adhesion signaling; mechanotransduction; survival regulation	FAK/Src activation; cytoskeletal tension	HNSCC, glioblastoma, breast cancer	Integrin inhibition; radiosensitization	[21, 22, 28, 38, 44, 79, 193, 194]
Focal adhesion signaling	FAK, Src, paxillin, p130CAS	Signal integration at focal adhesions	AKT/MAPK activation; migration; survival	Multiple solid tumors	FAK inhibitors; combination therapy	
Integrin-RTK crosstalk	EGFR, IGF-1R, VEGFR, PDGFR	Amplifies stress adaptation and repair signaling	PI3K/AKT, ERK signaling	ECM-rich tumors	Combined RTK/integrin targeting	[195–198]
Cytoskeletal mechanotrans-duction	RhoA/ROCK, actomyosin, ILK-PINCH-Parvin	Mechanical force transmission	Nuclear tension; chromatin regulation	Invasive and stiff tumors	ROCK/ILK targeting	[34, 62, 63, 151, 152]
Nuclear/ chromatin regulation	LINC complex, lamin A/C, HP1, BRD-associated chromatin regulators	DDR regulation; repair pathway modulation	NHEJ/HR balance; chromatin accessibility	3D ECM cultures	Chromatin-targeting radiosensitization	
Stress adaptation pathways	NF-κB, ROS buffering, YAP/TAZ, cGAS-STING	Adaptive survival under genotoxic stress	Inflammation; immune remodeling; stemness	Context-dependent	Combination therapies	[18, 20, 23, 24, 199]
Cell fate programs	Senescence, ferroptosis, lineage plasticity	Therapy adaptation and persistence	Tumor evolution; resistant states	Heterogeneous tumors	Synthetic lethality strategies	[200, 201]

As heterodimeric transmembrane receptors, integrins mediate cell-ECM interactions and organize signaling platforms at focal adhesions (FA). These structures, generally referred to as the adhesome [194, 195], integrate biochemical signals with mechanical inputs transmitted via the actin cytoskeleton, thereby tightly coupling extracellular cues to nuclear responses that govern survival, proliferation, and stress adaptation, including responses to ionizing radiation [22, 156, 196] (for further review see [38, 40, 202, 203]).

Efficient signal transduction frequently involves crosstalk between integrins and growth factor receptors (GFR), predominantly RTKs [22, 46]. This cooperation involves specific receptor pairings, shared adaptor proteins, and coordinated trafficking, forming a highly integrated signaling network that sustains tumor survival, proliferation, migration, invasion, and therapy resistance (Table 3) [197, 204]. Among integrins, β1 integrin serves as a central node that functionally, and in specific contexts physically, associates with multiple RTKs, including EGFR, PDGFRβ, and IGF-1R [197, 205]. Upon ECM engagement (e.g., fibronectin or collagen), β1 integrins undergo conformational activation and clustering, leading to recruitment of FAK and Src family kinases [202]. This promotes phosphorylation of adaptor proteins such as p130CAS, paxillin, Shc, and Nck, facilitating assembly of signaling complexes that enable ligand-independent activation of selected RTKs, most prominently EGFR.

In parallel, integrin activation stabilizes the integrin-linked kinase (ILK)-PINCH-parvin (IPP) complex, which serves as a scaffold linking integrins to the actin cytoskeleton and coordinating downstream signaling [41, 206]. ILK primarily functions as a structural adaptor that supports AKT pathway activation through protein-protein interactions rather than direct kinase activity [207]. PINCH1 is critical for complex stability and for recruitment of additional signaling components that regulate survival under stress conditions.

A prototypical example of integrin-RTK crosstalk is the α5β1 integrin-EGFR axis. Integrin engagement induces Src-dependent phosphorylation of EGFR in the absence of ligand, leading to EGFR monomer activation, facilitating EGFR homo- and heterodimerization and amplifying downstream RAS/RAF/MEK/ERK and PI3K/AKT signaling [208]. Conversely, EGFR activation promotes integrin activation via talin and kindlin recruitment and regulates integrin trafficking through Rab-dependent endosomal pathways, reinforcing adhesion signaling and sustaining receptor signaling output.

Distinct integrin-RTK modules operate in a context-dependent manner. αvβ3 integrin-VEGFR2 complexes, particularly in endothelial and glioblastoma cells, promote receptor clustering and modulate VEGFR2 trafficking, enhancing PLCγ-, PI3K/AKT-, and ERK-dependent signaling to drive angiogenesis and survival [209, 210]. Ionizing radiation can potentiate this axis by increasing VEGF availability and integrin expression, thereby reinforcing signaling through a feed-forward mechanism [211]. Similarly, αvβ5 integrin contributes to RTK signaling by regulating receptor localization and clustering [212, 213].

The α6β4 integrin-ErbB axis appears as a mechanistically distinct paradigm. Following phosphorylation of its cytoplasmic domain, α6β4 integrin disengages from hemidesmosomes and redistributes to signaling complexes, where it associates with ErbB receptors (EGFR/ErbB2) [214–216]. This promotes recruitment of Shc and activation of PI3K, as well as engagement of ILK-containing scaffolds, resulting in sustained PI3K/AKT signaling and enhanced receptor recycling, leading to prolonged signal duration and promotion of invasive and survival phenotypes.

Beyond direct receptor interactions, integrin-RTK crosstalk is organized by adhesome networks that integrate signaling and cytoskeletal dynamics. The IPP complex and adaptor proteins such as Nck1/2 couple phosphorylated receptors to actin remodeling, while integrin-dependent cytoskeletal tension-mediated by RhoA/ROCK signaling and actomyosin contractility modulates RTK signaling by regulating receptor clustering, membrane organization, and endocytic trafficking [46, 216]. Importantly, this contractile input is not restricted to adhesion receptors: G-protein-coupled receptors (GPCRs) sensing soluble microenvironmental cues converge on the same axis via Gα12/13-coupled RhoGEFs that activate RhoA, thereby reinforcing ROCK-driven actomyosin contractility. Acting in parallel, a RhoA-independent route, Ca²⁺/calmodulin-activated myosin light-chain kinase (MLCK), also phosphorylates the regulatory myosin light chain, so that ROCK and MLCK jointly set myosin II activity and the level of cytoskeletal tension fed back onto integrin-RTK signaling. These mechanisms enhance signaling robustness and plasticity. In line, AKT- and NF-κB-dependent transcriptional programs suppress apoptosis via upregulation of anti-apoptotic proteins, including BCL-2 family members and survivin [217–221]. Importantly, the network exhibits substantial redundancy: integrin-associated scaffolds like ILK-PINCH1 can sustain downstream signaling when RTK activity is inhibited, whereas RTK can compensate for impaired adhesion signaling [222–224]. In 3D ECM-rich environments, these interactions give rise to spatially organized signaling hubs, at which canonical signaling pathways are amplified and potentiate RTK activation in a ligand-independent manner, thereby increasing signaling efficiency and resistance to perturbation under genotoxic stress. These interactions form the core signaling architecture summarized in Figs. 2 and 3.

Collectively, integrin-RTK crosstalk represents a major mechanism by which the TME promotes adaptive therapy resistance. Its organization at membrane-proximal adhesion sites, integration with mechanotransduction, and inherent signaling redundancy underscore the need for combinatorial therapeutic strategies targeting integrins, RTK, and major scaffold components to effectively disrupt these networks and minimize compensatory signal transduction, enhancing treatment response. However, integrins are not the sole mechanosensitive regulators implicated in radiation response. Additional pathways involving Yes-associated protein 1/WW-domain-containing transcription regulator 1 (YAP/TAZ), myocardin-related transcription factors/serum response factor (SRF) signaling, caveolar dynamics, Piezo channels, and cytoskeletal remodeling likely cooperate with adhesion signaling in context-dependent manners [225–227]. Moreover, these pathways and processes are increasingly recognized to be shaped by additional context-dependent adaptive programs operating across metabolic, inflammatory, and lineage-regulatory networks in irradiated cells [199, 200, 228]. Radiation-induced cytosolic DNA sensing through the cGAS-STING pathway links DNA damage to inflammatory signaling and immune remodeling, influencing anti-tumor immunity as well as chronic pro-tumorigenic inflammation [201]. Additional adaptive mechanisms including metabolic rewiring, ROS/redox buffering, ferroptosis susceptibility, senescence-associated secretory phenotypes, and lineage plasticity further contribute to heterogeneous radiation responses across tumor ecosystems [198, 229]. Many of these processes are dynamically influenced by ECM architecture, hypoxia, stromal interactions, and mechanical stress, suggesting substantial functional crosstalk between classical adhesion signaling and broader stress-adaptation networks. Future studies will be required to define how these pathways interact across distinct tumor ecosystems.

### Cell adhesion-mediated radioresistance

CAM-RR describes a microenvironment-mediated survival mechanism in which interactions between tumor cells and the ECM attenuate radiation-induced cytotoxicity [81, 230]. These findings challenge the traditional view of radiosensitivity as a purely cell-intrinsic property. It emerges as an integrated systems-level phenotype defined by the coordinated activity of the components summarized in Figs. 2 and 3; Table 3.

Although initially identified in 2D culture systems, CAM-RR is markedly enhanced in 3D ECM contexts that more accurately recapitulate in vivo tumor architecture. Similar phenomena have been described for chemotherapeutic agents and targeted therapies in a variety of tumor types, including multiple myeloma, leukemia, prostate cancer, breast cancer, glioblastoma, pancreatic carcinoma, head and neck squamous cell carcinoma, hepatocellular carcinoma, lung cancer, and many others [217, 218, 231–244]. In these settings, integrin engagement promotes the formation of focal adhesion-based signaling platforms that integrate adhesion signaling with RTK activity, cytoskeletal dynamics, and nuclear stress responses (Figs. 2 and 3; Table 3).

Mechanistically, CAM-RR is driven by the coordinated activation of survival and repair pathways downstream of focal adhesions (Fig. 3). Key components, as described above, include a large variety of adhesome components such as FAK, ILK, PINCH1, Nck as well as PI3K/AKT and MAPK signaling [22, 38, 95, 202, 207, 217, 224, 231–234, 236, 245–267]. These pathways suppress apoptosis and promote clonogenic survival, viability, and stress adaptation in cells exposed to ionizing radiation.

Moreover, integrin-mediated adhesion and mechanotransduction, together with integrin-RTK crosstalk, form a tightly coupled regulatory network that controls the DDR and DSB repair efficiency and pathway choice [34, 94, 220, 221, 268–278]. Upon ECM engagement, integrins (particularly β1 and αv) assemble focal adhesion complexes and activate FAK/Src-dependent signaling that cooperates with RTKs such as EGFR and IGF-1R. This crosstalk amplifies downstream PI3K/AKT signaling and promotes activation of core DNA damage response kinases, including DNA-PKcs and ATM, thereby enhancing DSB repair capacity. Functionally, this signaling axis has been reported to preferentially support NHEJ by stabilizing DNA-PKcs activity, facilitating Ku70/80 recruitment, and promoting rapid end ligation. This has been demonstrated in studies showing that β1 integrin signaling and adhesion-dependent pathways are required for efficient NHEJ and that their disruption impairs repair and radiosensitizes tumor cells [34, 94, 279]. In parallel, integrin-driven cytoskeletal tension-transmitted via actomyosin contractility and the LINC complex modulates nuclear architecture and chromatin compaction, which critically determines DNA end accessibility. Mechanically stabilized chromatin states may limit DNA end resection and thereby shifting the balance from HR to NHEJ context-dependently. In contrast, loss of adhesion or reduced mechanotransduction leads to chromatin relaxation, enhanced resection, and a relative shift toward HR [280, 281]. Integrin-RTK cooperation further enhances this balance by sustaining AKT- and ERK-dependent signaling that regulates the localization and activity of repair factors, including DNA-PKcs, BRCA1, and RAD51, integrating biochemical signaling with chromatin-based control of repair pathway utilization. Together, these findings support a model in which DSB repair is dynamically influenced by adhesion-dependent signaling networks and integrin-RTK interactions, which bias repair toward rapid, pro-survival NHEJ while constraining HR in mechanically defined tumor microenvironments. Figures 2 and 3 as well as Table 3 summarize these findings in a functional map of integrin-driven radioresistance including components that define a conserved CAM-RR signaling axis.

Of translational interest was and still is whether integrin targeting is effective in chemo- and radiosensitization. Intriguingly, experimental disruption of integrin signaling, either through genetic approaches or functional blocking antibodies against integrin subunits, consistently sensitizes tumor cells to radiation (and other cytotoxic, genotoxic and molecular agents) in 3D tumor models and in vivo models, thus underscoring the causal role of adhesion signaling in therapy resistance [38, 282–285]. Since particularly β1- and αv-integrin family members are key mediators of CAM-RR, inhibition of these integrins was of interest. Strong preclinical evidence showed that β1 integrin blockade sensitizes tumors to irradiation by impairing DNA repair and promoting apoptosis [231, 232, 286], while αvβ3/αvβ5 inhibition with cilengitide improves radiotherapy response and reduces tumor growth in vivo [287–290]. However, clinical translation has been limited: the Phase III CENTRIC trial in glioblastoma showed no survival benefit for cilengitide combined with radiochemotherapy, despite earlier studies of efficacy in selected subgroups [291, 292]. Similarly, β1 integrin targeting approaches remain largely preclinical, and downstream FAK inhibitors (e.g., defactinib) have demonstrated limited single-agent activity but potential in combination settings [293, 294]. It needs to be emphasized here that integrin inhibition per se is attractive but clinical trials require patient selection and biomarkers as well as consideration of other sequences between drug application and radiotherapy, among various additional factors.

These discrepancies in effectiveness likely reflect the redundancy and plasticity of adhesion-associated signaling networks, as well as tumor heterogeneity and microenvironmental complexity [236, 295, 296]. The disconnect between robust preclinical radiosensitization and the clinical failure of agents like cilengitide highlights a critical translational gap. Future clinical success likely hinges on moving away from “one-size-fits-all” integrin inhibition toward biomarker-stratified cohorts. For instance, advanced 3D models such as PDOs can now be used as ex vivo avatars to pre-screen patients for high integrin-RTK signaling signatures, ensuring that only those most likely to benefit from combinatorial targeting are enrolled in trials. A critical unresolved question is whether integrin signaling represents a universal driver of radioresistance or a context-dependent amplifier of pre-existing survival programs. While extensive preclinical evidence supports a causal role in ECM-rich environments, clinical failures such as those observed for cilengitide suggest that integrin targeting alone is insufficient in heterogeneous tumor settings. This raises the possibility that integrin signaling primarily functions as a network hub that stabilizes multiple redundant pathways rather than acting as a single dominant driver. When this is considered more broadly in the context of the entire matrisome, pan-cancer analyses clearly showed that matrisome alterations are highly prevalent, affecting up to ~ 33.5% of tumors for integrin genes alone, with broader ECM-related gene sets exhibiting mutation or copy number variation frequencies ranging from 1% to 80% [297–303]. Individual matrisome genes are infrequently mutated (~ 1–3%), suggesting network-level dysregulations rather than classical oncogene-driven mutation patterns. Collagen genes, despite being mutated in ~ 60% of hereditary ECM disorders, are more commonly transcriptionally upregulated in cancer than other matrisomal proteins, with fold changes typically ranging from 1.4 to 1.9 but exerting profound effects on tumor stiffness, immune exclusion, and signaling. Consequently, future strategies must address not only integrins themselves but also other matrisomal components and their integration with and co-targeting of RTK signaling, cytoskeletal dynamics, and/or chromatin regulation.

Overall, CAM-RR demonstrates a paradigmatic example of microenvironment-driven therapy resistance, in which integrin signaling networks integrate biochemical and mechanical cues to sustain tumor cell survival under genotoxic stress.

### Pros and cons of 3D tumor models

The aspects mentioned above offer a repertoire of considerations for the optimal selection of a 3D tumor model that best corresponds to the tissue context and enables the answering of the respective scientific, translational question (see Fig. 1; Tables 1 and 2). 3D tumor models have become indispensable for studying radiation response, as they better recapitulate key aspects of tumor architecture and microenvironmental gradients than conventional 2D systems. Model-specific strengths (Table 1) determine which resistance mechanisms and selected tumor biology features can be captured, guiding the interpretation of experimental outcomes (Tables 1 and 2). As detailed in Table 2, the choice of a 3D platform dictates the available experimental readouts.

#### Multicellular tumor spheroids 

Are among the most widely used 3D systems due to their high reproducibility, scalability, and compatibility with medium- to high-throughput workflows [69, 70, 72]. They reliably establish physiologically relevant gradients of oxygen, nutrients, pH, and proliferation, thereby enabling the study of hypoxia-driven radioresistance and drug penetration. Their relatively simple and standardized formation allows systematic comparisons across experimental conditions. However, spheroids lack a defined ECM architecture and typically do not incorporate stromal or immune components, limiting their ability to model adhesion-dependent signaling networks in a physiologically relevant context. In addition, their structural simplicity may underestimate the spatial and mechanical heterogeneity present in vivo. 

#### ECM-based 3D culture systems

Including hydrogels such as collagen, Matrigel, or synthetic matrices, partially overcome these limitations by restoring cell-matrix interactions and enabling investigation of mechanotransduction and matrix-dependent signaling [55, 56, 66, 75, 76, 101]. These systems are particularly valuable for studying how ECM composition, stiffness, and architecture influence radiation response, DNA repair, and cell survival. They also allow controlled manipulation of individual matrix parameters. However, naturally derived matrices often suffer from batch-to-batch variability and undefined composition, which can affect reproducibility. Synthetic matrices provide greater control but may lack biological complexity. Furthermore, many ECM-based systems still do not fully incorporate the cellular diversity of the TME. 

#### Patient-derived organoids (PDO)

Represent a major advance in translational cancer research, as they preserve major features of the original tumor, including genetic heterogeneity, clonal architecture, and, to some extent, phenotypic diversity [50, 73, 77, 304]. This makes them highly suitable for personalized therapy testing and for studying inter-patient variability in response to radiation, chemotherapy, and chemoradiation. PDOs have shown promise in predicting clinical treatment outcomes, including radiosensitivity. However, they are resource-intensive, require specialized expertise, and are less amenable to large-scale screening. Standard organoid cultures often lack components of the TME such as vasculature, immune cells, and fibroblasts, which are critical regulators of radiation response. Long-term culture may also introduce selection biases and drift. To address these limitations, organoid-based assembloids have been developed, in which tumor organoids are either co-cultured alone or with additional cell types [107–110]. While assembloids significantly enhance physiological relevance and provide new opportunities to study microenvironment-driven radio(chemo)resistance and radio-immunological effects, they also introduce increased technical complexity, reduced standardization, and challenges in reproducibility and scalability. 

#### Co-culture systems 

aim to address these limitations by incorporating additional cell types, including CAFs, endothelial cells, and immune cells [74, 305, 306]. These models enable the study of cell-cell interactions, paracrine signaling, and microenvironment-mediated resistance mechanisms, including those affecting radiation response and repair processes. For example, fibroblast-mediated ECM remodeling or immune-mediated cytokine signaling can significantly alter treatment sensitivity. However, increasing biological complexity comes at the cost of reduced experimental control and reproducibility. The relative proportions, activation states, and spatial organization of different cell types can vary substantially, complicating data interpretation and standardization across studies.

In strictly mechanobiological terms, these platforms differ in how faithfully they reconstitute the adhesion cues that drive CAM-RR. Standard PDOs are propagated in soft basement-membrane extract and therefore capture genetic and epithelial heterogeneity far better than tumor-relevant stiffness, fibrillar collagen architecture, or integrin-dependent stromal signaling; applying defined mechanoregulation generally requires embedding them in tunable matrices or in assembloids and co-culture formats. Co-culture systems make the stromal contribution explicit: CAFs deposit, crosslink, and align collagen, stiffen the matrix, and thereby amplify integrin-FAK and YAP/TAZ mechanotransduction in adjacent tumor cells [45, 147], a configuration increasingly used to model adhesion-dependent radio(chemo)resistance in engineered stromal tissues [84, 307, 308].

Advanced 3D platforms, including bioprinted tumor constructs and microfluidic tumor-on-a-chip systems, offer control over spatial organization, architecture, and dynamic processes [85–90, 309–312]. Bioprinting enables defined placement of cell types and matrix components, while microfluidic systems permit controlled perfusion and integration of vascular-like structures, making both well suited to modeling temporal treatment responses. They remain technically demanding, infrastructure-dependent, and limited in scalability as well as standardization.

Two caveats deserve emphasis for mechanobiological work. First, the tunability of bioprinting is constrained by available bioinks, which often fail to reproduce native ECM architecture and mechanics and are in several cases intrinsically pro-inflammatory, potentially confounding radiation read-outs [91, 311, 313, 314]. Second, a complementary class of scaffold-free, self-assembled engineered tissues, in which stromal cells produce and organize their own ECM; an approach derived from autologous skin substitutes developed for burn patients, can reconstruct tissue-relevant architecture, composition, and stiffness, including CAF-driven stiffening, offering a tunable, biomaterial-free platform for mechanotransduction-dependent hypotheses [84, 307, 308].

Beyond model-specific considerations, several cross-cutting challenges remain. These include the limited representation of functional vasculature and immune surveillance, difficulties in modeling long-term treatment responses and tumor evolution, and variability in readouts across platforms. In addition, differences in culture conditions, oxygen control, and irradiation protocols can significantly influence experimental outcomes and complicate comparisons between studies.

No single model captures tumor complexity, so integrative strategies combining complementary systems are increasingly favored: spheroids or ECM-based models for mechanistic perturbations, and PDO, slice cultures, co-culture systems or even patient-derived xenografts (PDX) for validation in clinically relevant contexts [315]. Model choice should follow the biological question rather than assumptions that more complex systems are universally superior. Computational analysis is a useful adjunct here: machine learning models, trained on high-content imaging data from 3D systems can quantify radiation-induced spatial and morphological changes more reproducibly than manual scoring, and in silico screening can prioritize radiation-drug combinations for experimental testing. Such approaches accelerate hypothesis generation but do not substitute for biological validation, and their value is bounded by the quality and standardization of the underlying 3D data.

### Translational applications

The integration of 3D tumor models into radiation oncology is redefining translational research by enabling functional interrogation of microenvironment-driven therapy resistance. Unlike conventional 2D systems, 3D models preserve key features of tumor architecture, ECM composition, and spatial signaling organization, providing access to resistance mechanisms - such as CAM-RR - that become even more evident in physiologically relevant contexts.

A central implication is that radiosensitivity can be assessed as a functional, context-dependent phenotype rather than a purely molecular signature. PDO and advanced co-culture systems enable ex vivo testing of radiation response under conditions that retain tumor heterogeneity and, to varying degrees, microenvironmental signaling. These platforms therefore offer a foundation for functional precision radiotherapy, in which treatment decisions are informed by experimentally measured response profiles rather than inferred from genomic alterations alone.

Beyond response prediction, 3D models provide a mechanistic framework for biomarker discovery linked to microenvironmental dependencies. Integrin expression patterns, focal adhesion components, and ECM signatures like collagen organization or fibronectin deposition can serve as candidate biomarkers of adhesion-driven resistance. By systematically interrogating these parameters across model systems (Tables 1 and 2; Fig. 1), it becomes possible to identify context-specific vulnerabilities and to stratify tumors according to their reliance on integrin-centered signaling networks. Such biomarkers may complement existing molecular classifiers by incorporating spatial and biomechanical dimensions of tumor biology.

The translational potential of these models is further enhanced by their compatibility with targeted intervention strategies. Preclinical data indicate that disruption of integrin signaling (e.g., β1 integrin), FAK/Src, or associated scaffold proteins (ILK-PINCH1) can sensitize tumors to irradiation, particularly in ECM-rich environments where CAM-RR is dominant (reviewed in [22, 38, 316]). However, as highlighted by clinical studies, single-agent targeting is often insufficient due to signaling redundancy within integrin-RTK networks. This also highlights an important limitation of current precision oncology approaches, which often rely on genomic alterations without accounting for microenvironmental dependencies. Integrin-mediated signaling, as a context-dependent regulator, exemplifies mechanisms that cannot be inferred from genomic data alone and therefore require functional assessment in physiologically relevant models. 3D systems provide a unique platform to rationally design and test combination therapies that simultaneously target adhesion signaling, RTKs, and downstream survival pathways, thereby overcoming compensatory mechanisms. Several clinical studies have evaluated therapeutic strategies targeting integrins, focal adhesion signaling, and broader matrisome-associated pathways across multiple tumor entities ([192, 206, 237, 283, 291, 316–324]; www.clinicaltrials.gov). Collectively, these trials illustrate both the translational potential and the biological challenges associated with disrupting highly adaptive adhesion-dependent signaling networks in cancer. Importantly, the limited efficacy observed in several early integrin-targeting trials likely reflects the redundancy and compensatory plasticity of matrisome-associated signaling networks rather than the absence of biological relevance. These findings support the concept that effective targeting of adhesion-dependent radioresistance will likely require biomarker-guided patient stratification and combinatorial therapeutic approaches against RTKs, immune components or others.

To keep this message radiation-specific, radiosensitization should be distinguished from broader therapy resistance. Direct evidence that perturbing the ECM-integrin axis modulates radiosensitivity comes from studies in which β1 integrin inhibition impaired DSB repair and radiosensitized 3D head and neck and other models, FAK/Src disruption enhanced radiation response, and αvβ3/αvβ5 integrin blockade with cilengitide augmented radiotherapy in vivo, while CAF-mediated stiffening conversely conferred radioprotection that ECM normalization relieved [38, 94, 231, 232, 287, 318]. Mechanisms invoked elsewhere, such as chemoresistance, immune evasion, and invasion, are not radiation-specific; we therefore restrict translational claims to settings with a measured radiation end point, such as clonogenic survival, DSB repair kinetics, or in vivo tumor control after irradiation.

In addition to direct integrin and focal adhesion targeting, a broader spectrum of matrisome-directed therapeutic strategies is increasingly emerging. Anti-fibrotic approaches targeting TGFβ signaling aim to reduce ECM deposition, stromal activation, and therapy-induced desmoplasia, thereby improving tissue perfusion and immune accessibility [325, 326]. Similarly, inhibition of LOX family enzymes, which mediate collagen crosslinking and ECM stiffening, has shown potential to interfere with mechanotransduction-dependent survival signaling and metastatic niche formation [327]. Additional approaches include targeting discoidin domain receptors (DDR1/2), collagen-sensing receptor tyrosine kinases implicated in invasion, immune evasion, and therapy resistance [328–330]. Mechanosensitive transcriptional regulators such as YAP/TAZ are also attracting increasing interest because of their central role in translating ECM stiffness and cytoskeletal tension into adaptive transcriptional programs promoting stemness, survival, and radioresistance [45]. Furthermore, therapeutic modulation of CAFs, collagen remodeling, and ECM-normalization strategies seek to reprogram tumor architecture rather than directly eliminate tumor cells, thereby potentially enhancing drug delivery, immune infiltration, and radiation response. Altogether, these approaches highlight the matrisome as a therapeutically actionable ecosystem whose modulation may complement conventional cytotoxic and molecular therapies within multimodal precision oncology.

Mechanistically, stromal stiffening is sustained by a feed-forward circuit in which tumor cells, CAFs, and tumor-associated macrophages reciprocally reinforce ECM deposition and crosslinking. Because this cellular network, rather than any single matrix gene, drives the stiff phenotype, disrupting the circuit, for example by reprogramming CAFs or macrophages, offers an indirect route to soften the matrix, relieve mechanotransduction-dependent radioresistance, and improve perfusion and immune access [331]. Resolving how this loop shapes radiation response will require 3D co-culture and assembloid systems able to sustain tumor-stroma-immune crosstalk over the weeks-long timescale on which stiffening develops.

Beyond cell-intrinsic resistance, the interaction between radiotherapy and antitumor immunity is increasingly relevant. Radiation can promote immunogenic cell death and release of neoantigens and damage-associated molecular patterns (DAMPs) [78, 332]. However, this is frequently compromised by the immunosuppressive and physically restrictive architecture of the TME. Functional 3D models, particularly PDO co-cultures with autologous immune cells [79, 80, 333], are well suited to dissect these dynamics, including adhesion-mediated immune exclusion in which a dense, stiff ECM and integrin-dependent signaling act as barriers to T-cell infiltration after irradiation [334, 335]. Such models have helped nominate targets, including the ILK/FAK axis (Table 3), whose modulation may sensitize poorly infiltrated tumors to radio-immunotherapy, although this remains to be tested prospectively.

An additional key opportunity lies in the integration of 3D model-derived insights with non-invasive clinical imaging and computational approaches. Techniques such as MRE enable in vivo assessment of tissue stiffness, a surrogate for ECM composition and remodeling [154, 155, 336, 337], while radiomics can capture spatial heterogeneity in tumor structure [338, 339]. Coupling these modalities with functional data from 3D models may allow the development of imaging-informed biomarkers that reflect underlying adhesion and mechanotransduction states and beyond. This integration could support dynamic monitoring of tumor evolution during therapy and enable adaptive treatment strategies.

3D models also provide a platform to evaluate emerging modalities such as particle therapy and FLASH radiotherapy, whose biological effectiveness depends on oxygenation, tissue architecture, and microenvironmental context. By reconstituting these parameters, organoids, assembloids, co-cultures, and microfluidic systems allow mechanistic dissection of modality-specific responses and identification of patient subgroups most likely to benefit.

Taken together, 3D models move translational radiobiology from a reductionist toward a functional, network-level approach. By resolving spatially organized signaling and its contribution to resistance, they expose actionable vulnerabilities, support biomarker-guided patient stratification, and enable rational combination design with integrin-related adhesion signaling emerging as a unifying axis linking TME, DNA repair, and survival.

### Challenges and future directions

Despite significant progress, several challenges must be addressed to fully realize the translational potential of 3D tumor models in radiation oncology. A major limitation is the lack of standardization across model systems, including variability in ECM composition, oxygen and nutrient supply, culture conditions in general, and analytical endpoints, which complicates reproducibility and cross-study comparisons. Most current models still incompletely recapitulate critical aspects of the TME like vascularization, immune dynamics, and long-range stromal interactions, limiting their predictive accuracy. Another important challenge lies in distinguishing biologically meaningful emergent properties from model-specific artifacts introduced by engineered culture systems, matrix compositions, or artificial growth conditions. Furthermore, integration of complex 3D systems into clinically relevant timelines remains challenging, particularly for patient-derived models intended to inform treatment decisions. From a technological perspective, scalability, cost, and accessibility continue to constrain widespread adoption, especially for advanced platforms such as bioprinting and microfluidic systems.

A prerequisite for such validation, which deserves emphasis at the outset, is systematic benchmarking of existing models against the biology and temporal evolution of the tumors they represent. At present it is largely unknown whether widely used systems capture the relevant biology, because comparative, ground-truth data are scarce; indeed, much of the difficulty in standardization stems from the fact that the mechanical and compositional parameters that actually need to be modeled have not been well characterized across the tumor spectrum.

Future efforts should focus on the development of standardized, modular platforms that combine multiple microenvironmental components and can be integrated with high-content imaging, single-cell sequencing, and computational modeling. Such approaches will be essential to extract clinically meaningful information from these complex datasets. Importantly, prospective clinical studies are required to validate 3D model-derived biomarkers and to establish their utility in guiding treatment decisions. Ultimately, the convergence of advanced tumor modeling, molecular profiling, and innovative radiation technologies holds the promise of transforming radiation oncology from a largely uniform treatment approach to a precision discipline tailored to the biological and biomechanical properties of individual tumors.

## Conclusions

The transition from 2D to 3D modeling is a conceptual shift toward understanding cancer as an architecture-dependent disease. The central message of this review is that radiation response is governed by a continuous mechanotransduction axis in which ECM composition and stiffness are sensed by integrins and transmitted through focal adhesions, the cytoskeleton, and the LINC complex to the nucleus, where they are converted into chromatin states that set DSB-repair capacity and pathway choice. CAM-RR is the integrated output of this axis rather than the property of any single node. Because the axis is robust and redundant, durable radiosensitization will require co-targeting of its mechanical and biochemical components, guided by functional 3D models and non-invasive mechanical biomarkers. This framework is useful only if it is falsifiable, and it makes several testable predictions. First, matched tumor cells cultured across a defined stiffness range should show a graded shift in DSB repair pathway choice toward NHEJ as stiffness increases, measurable by repair-reporter assays in tunable hydrogels. Second, CAM-RR should be recapitulated in matrix-embedded and engineered tissues but not in scaffold-free spheroids or 2D, providing a direct test of which platforms capture the phenotype. Third, normalizing ECM stiffness, whether by lysyl-oxidase inhibition, CAF reprogramming, or matrix-tuned culture, should radiosensitize to a degree predicted by the baseline stiffness of the model. Fourth, mechanical biomarkers such as MRE-derived stiffness should correlate with adhesion-dependent radioresistance signatures and, ultimately, with clinical radiation response. Testing these predictions in standardized, benchmarked 3D systems is, in our view, the most direct route from the mechanistic picture assembled here to microenvironment-aware radiotherapy.

## Data Availability

No datasets were generated or analysed during the current study.

## Abbreviations

2D: Two-dimensional
3D: Three-dimensional
AI: Artificial intelligence
ATM: Ataxia-Telangiectasia Mutated
BANF: Barrier To Autointegration Factor
CAF: Cancer-associated fibroblast
CAM-RR: Cell adhesion-mediated radioresistance
DDR: DNA damage response
DDR1/2: Discoidin Domain Receptor 1/2
DNA-PKcs: DNA-dependent Protein kinase catalytic subunit
DSB: Double stranded break
ECM: Extracellular matrix
EGFR: Epidermal growth factor receptor
ErbB: Erythroblastic leukemia viral oncogene B
FA: Focal adhesion
FAK: Focal adhesion kinase
GFR: Growth factor receptor
HP1: Heterochromatin protein 1
IGF-1R: Insulin-like growth factor receptor
ILK: Integrin-linked kinase
IPP: ILK-PINCH-Parvin complex
LEM: Lamina-associated polypeptide (LAP)2, emerin, and MAN1
LINC: Linker of nucleoskeleton and cytoskeleton
MRE: Magnetic resonance elastography
NHEJ: Non-homologous Endjoining
PDGFR: Platelet-derived growth factor receptor
PDO: Patient-derived organoid
PDX: Patient-derived xenograft
PINCH: Particularly Interesting New Cysteine-Histidine-rich protein
RTK: Receptor tyrosine kinase
TME: Tumor microenvironment
VEGFR: Vascular endothelial growth factor receptor
